# Elucidating Consequences of Selenium Crystallinity on Its Electrochemical Reduction in Aluminum–Selenium Batteries

**DOI:** 10.1021/acsmaterialslett.4c00531

**Published:** 2024-05-24

**Authors:** Leo W. Gordon, Rahul Jay, Ankur L. Jadhav, Snehal S. Bhalekar, Robert J. Messinger

**Affiliations:** Department of Chemical Engineering, The City College of New York, CUNY, 160 Convent Ave., New York, New York 10031, United States

## Abstract

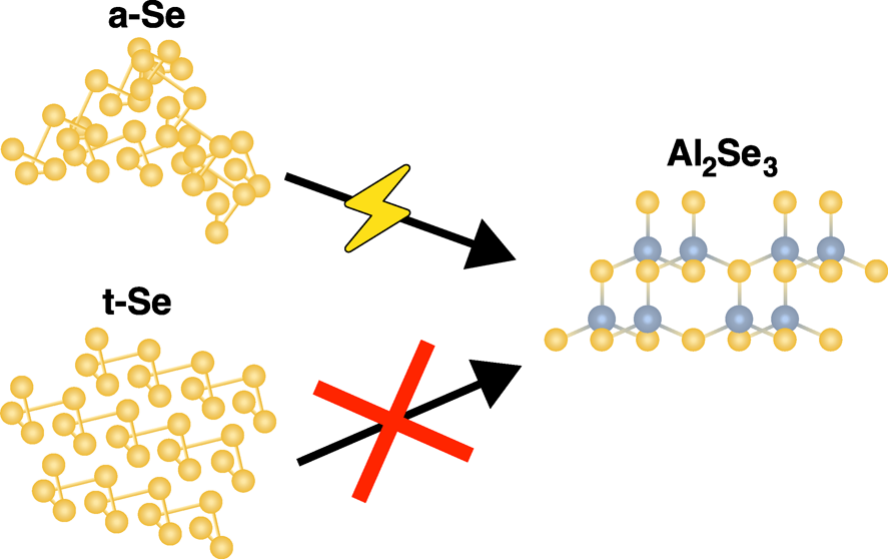

Selenium (Se) is an attractive positive electrode material for rechargeable aluminum (Al) batteries due to its high theoretical capacity of 2037 mA h g^–1^ and its higher electronic conductivity compared to sulfur. Selenium can undergo a series of electrochemical reactions between Se(–II) and Se(IV), resulting in a six-electron capacity per Se atom. However, existing Al–Se battery literature is inconsistent regarding the different electrochemical reactions possible, while the conditions enabling the electrochemical reduction of Se to Al_2_Se_3_ are not well understood. Here, we demonstrate that this electrochemical reduction is achievable using amorphous selenium but is suppressed for crystalline selenium. We further show that the electrochemical oxidation of Se to SeCl_4_, which occurs at higher potentials, reduces the long-range order of crystalline Se and enables its discharge to Al_2_Se_3_. Solid-state ^77^Se nuclear magnetic resonance (NMR) measurements further establish that the local Se helical structures are maintained upon the loss of crystallinity.

The nascent field of rechargeable aluminum (Al) batteries has generated promising results as a consequence of aluminum’s large theoretical capacity (2980 mA h g^–1^), high abundance, low cost, and inherent safety.^1,2^ Despite this promise, rechargeable aluminum batteries are currently hindered by a lack of high-capacity cathode materials, which furthermore must be compatible with chloroaluminate electrolytes.^3^ Elemental chalcogen electrodes such as sulfur (S) and selenium (Se) have garnered recent interest due to their large theoretical capacities;^2,4−7^ however, these conversion electrodes typically suffer from slow electrochemical kinetics, large volume changes upon cycling, and poor reversibility due to the formation of electrolyte-soluble reaction intermediates.^8^ Sulfur electrodes are highly resistive, necessitating the use of electrodes with high carbon content, which reduces their specific capacity. Conversely, selenium has a conductivity of 1.0 mS m^–1^, 25 orders of magnitude greater than that of sulfur (5.0 × 10^–25^ mS m^–1^), leading to smaller overpotentials under equivalent conditions.^9^

While sulfur electrodes in Al–S batteries have been well documented to undergo a two-electron reduction from elemental S to Al_2_S_3_, the analogous reaction of Se to Al_2_Se_3_ has thus far been inconsistently reported.^10−12^ To date, only a handful of papers on Al–Se batteries have been published,^6,9,13−20^ with some reporting only the electrochemical reduction reactions of selenium^6,9,13^ and others reporting only the electrochemical oxidation reactions of selenium.^15−20^ Thus far, only Zhang et al.^14^ has demonstrated the full 6-electron capacity of selenium, utilizing both the electrochemical reduction and oxidation reactions of Se. Interestingly, researchers that do not report the Se(0) to Se(−II) reduction reaction have used crystalline selenium electrodes.^15−18^ To investigate the disparity in reported electrochemical reaction mechanisms, we study, for the first time, the impact of selenium electrode crystallinity on the viability of the electrochemical reduction of Se to Al_2_Se_3_. Although they are both chalcogens, sulfur and selenium have significantly different structures, affecting both their local environments and packing arrangements. Sulfur is found as eight-membered puckered rings, whereas the stable allotrope of selenium has a trigonal helix structure; these structural differences have a profound effect on their respective behavior as electrode materials. Here, we prepared selenium electrodes using (i) crystalline trigonal selenium (*t*-Se) and (ii) amorphous selenium (*a*-Se) to understand how the electrode structure affects the electrochemical reactions that are possible. Al–Se batteries were studied using galvanostatic cycling and cyclic voltammetry, while the crystalline and local structures were determined by powder X-ray diffraction (XRD) and solid-state ^77^Se nuclear magnetic resonance (NMR) spectroscopy, respectively.

Cyclic voltammetry (CV) experiments using different voltage windows were performed on Al–Se cells by using either crystalline or amorphous Se electrodes to compare their electrochemical responses (Figure 1a,b). A CV conducted between 0.2 and 1.5 V vs Al/Al(III) (Figure 1a) using an *a*-Se electrode reveals the electrochemical reduction of Se to Al_2_Se_3_ (Se(0) to Se(−II)) during the reductive sweep, which is reversible upon the oxidative sweep. This electrochemical reaction is suppressed when using a crystalline *t*-Se electrode. Thus, elemental Se is only appreciably electrochemically reduced to Al_2_Se_3_ when using *a*-Se electrodes.

**Figure 1:**
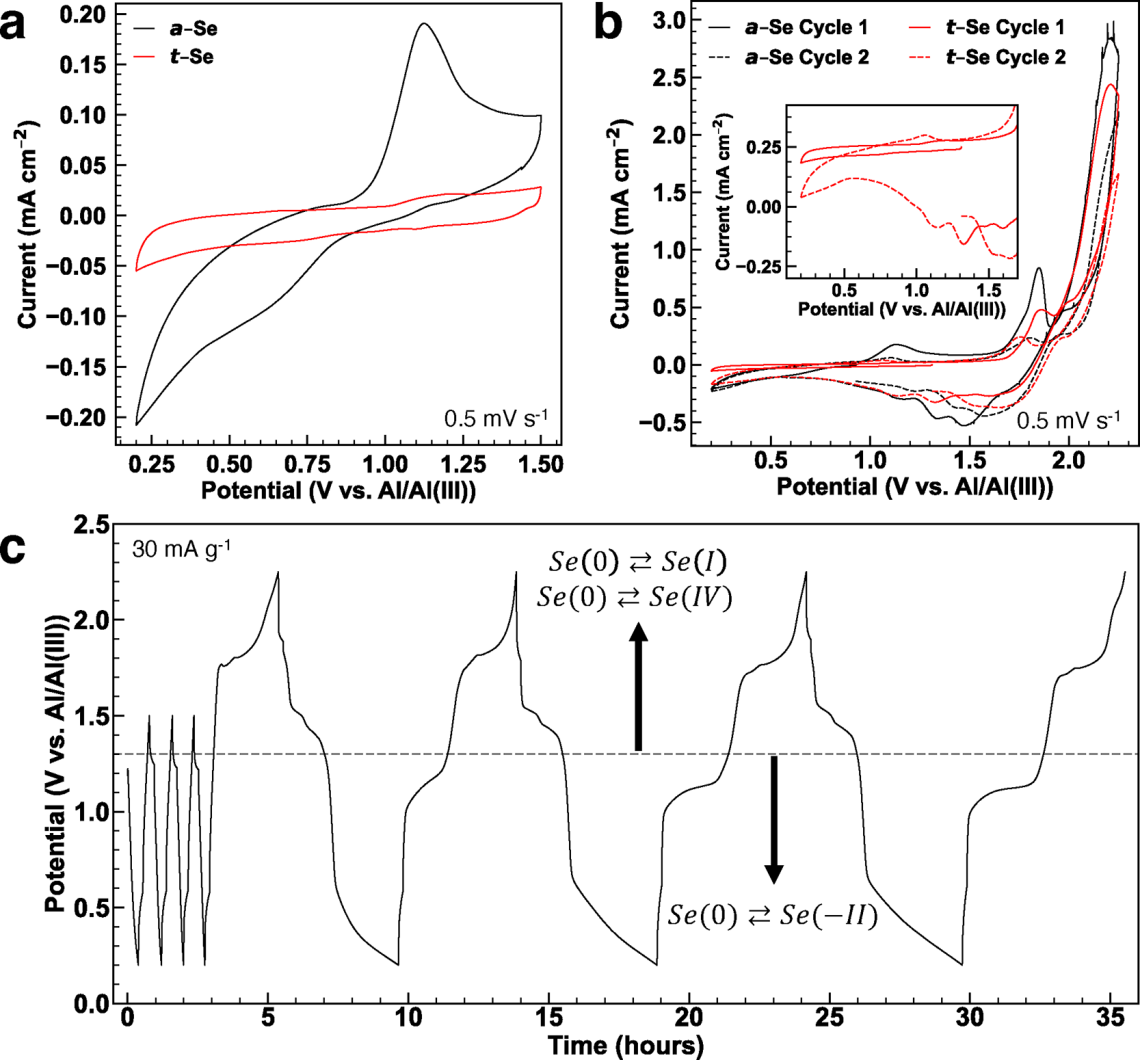
(a) Cyclic voltammetry of Al–Se cells using amorphous selenium (*a*-Se, black) or crystalline selenium (*t*-Se, red) electrodes with a potential window of 0.2–1.5 V (0.5 mV s^–1^, cycle 5). (b) Cyclic voltammetry of Al–Se batteries using *a*-Se (black) or *t*-Se (red) electrodes with a potential window of 0.2–2.25 V (0.5 mV s^–1^). Inset: 0.2–1.7 V region of the *t*-Se voltammograms showing emergence of a new reduction reaction following oxidation to 2.25 V. (c) Galvanostatic cycling of an Al–Se battery using a *t*-Se electrode (30 mA g^–1^). The region associated with reduction of selenium lies below the dashed line, and the region associated with oxidation of selenium lies above the dashed line.

When the upper potential range of the CV is extended from 1.5 to 2.25 V (Figure 1b), oxidation reactions occur at 1.75, 1.85, and 1.98 V that correspond to the oxidation of Se to selenium chlorides, including Se_2_Cl_2_^14−16,18^ (Se(0) to Se(I)) and SeCl_4_^14,15,17,18^ (Se(0) to Se(IV)). On the reductive sweep, the higher potential peaks between 1.0 and 1.75 V are associated with the electrochemical reduction of the selenium chlorides to elemental selenium, while the lower potential peak at approximately 0.5 V corresponds to the reduction of Se to Al_2_Se_3_, which interestingly is seen for both *t*-Se and *a*-Se electrodes.

At higher potentials, selenium undergoes oxidation to selenium chlorides, but the reverse reaction back to selenium is expected to result in a less crystalline Se structure. This change in structure is demonstrated by the fact that *t*-Se is not reduced to Al_2_Se_3_ during the initial reductive sweep of the voltammogram; this reduction is observed, however, with *t*-Se electrodes once the reversible oxidation of selenium has occurred (Figure 1b). This result confirms the finding from Figure 1a that crystallinity is a significant factor in achieving the electrochemical reduction of Se to Al_2_Se_3_.

The exclusive achievement of the Se to Al_2_Se_3_ electrochemical reduction with noncrystalline electrodes also held true for Al–Se cells under galvanostatic operation (Figure 1c). When Al–Se cells using *t*-Se electrodes are galvanostatically cycled using an upper voltage limit of 1.5 V, where the reversible Se to Al_2_Se_3_ reactions (Se(0) to Se(−II)) are expected, no such reactions are observed over multiple cycles. However, when the voltage limit is increased to 2.25 V, selenium is electrochemically oxidized to selenium chlorides (Se(0) to Se(I) and Se(IV)). Crucially, upon subsequent discharges, the elemental selenium crystallinity is reduced, as previously discussed. As a result, the conversion of Se to Al_2_Se_3_ becomes accessible upon subsequent cycles, thereby significantly increasing the specific capacity of the Al–Se cell (Figure 1c). The specific capacity associated with electrochemical selenium reduction increases for several cycles but is ultimately in competition with material dissolution into the electrolyte, which results in specific capacity fade (Figure S1 and the Supporting Information).

Powder X-ray diffraction measurements were performed to establish the crystallinity of the Se powders, electrodes, and cycled electrodes. The crystalline *t*-Se powder exhibits a hexagonally packed helical chain structure, often denoted as Se_∞_ on account of these indefinite helices.^21^ The diffraction pattern of *t*-Se (*t*-Se powder, Figure 2a) matches the expected pattern documented in the literature^22^ that is linked with the hexagonally packed trigonal chain structure (Figure 2a, inset). We anticipated that the melt-infused Se powder would result in the absence of the long-range order that is observed for *t*-Se. The diffraction pattern of *a*-Se powder (*a*-Se, Figure 2a) shows only broad, featureless reflections associated with the amorphous material. From these diffraction patterns, it is clear that the process of melting selenium eliminates the long-range crystalline order in selenium, yielding a noncrystalline starting material for electrode fabrication.

**Figure 2:**
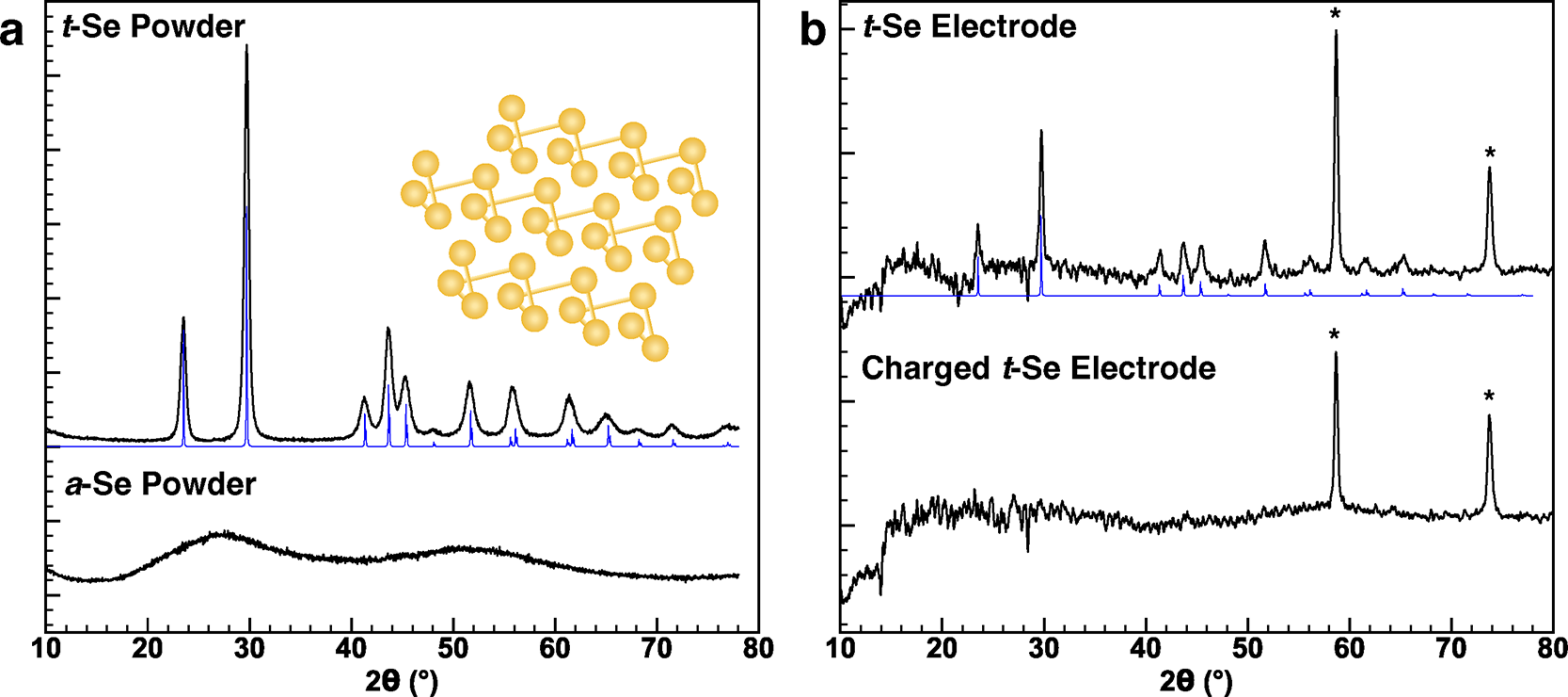
Powder XRD patterns of (a) *t*-Se powder (top) and *a*-Se melted into Ketjen black carbon (bottom). The calculated diffraction pattern of *t*-Se is overlaid in blue beneath the experimental pattern. Inset: *t*-Se crystal structure. (b) X-ray diffraction patterns from an uncycled *t*-Se electrode (top) and a *t*-Se electrode charged to 2.25 V (bottom). The background signal from Kapton tape in (b) has been subtracted (see Figure S7 for original XRD patterns and accompanying electrochemistry), and reflections from the Mo current collector are marked with asterisks (*).

To further compare the *a*-Se and *t*-Se electrodes, we melted selenium into the Ketjen black conductive carbon using an identical process for *a*-Se, but followed by an annealing step at 150 °C for 12 h in an attempt to recrystallize the Se. Powder XRD (Figure S2) and solid-state ^77^Se NMR (Figures S3 and S4) measurements establish that the selenium is a mixture of *a*-Se (51.5 mol %) and *t*-Se (49.5 mol %). Galvanostatic cycling of an Al–Se cell using an electrode prepared with this melt-annealed Se exhibits a specific capacity in the potential region due to the Se(0) to Se(−II) reaction, an expected consequence of the amorphous selenium still present (Figure S5). However, after undergoing the reversible selenium oxidation reactions, the specific capacity of the Se(0) to Se(−II) reaction increases, consistent with the conversion of crystalline to amorphous selenium. Note that the melt process reduces the primary particle size, as observed by SEM for both the melted and melt-annealed composite Se powders (Figure S6). These results indicate that the key driver for reducing Se to Al_2_Se_3_ is the crystallinity of selenium, as opposed to the particle size.

The loss of selenium crystallinity was also observed in powder XRD by comparing a pristine *t*-Se electrode with one charged to 2.25 V in an Al–Se cell (Figure 2b). The intensities of all *t*-Se reflections are diminished significantly, and only reflections corresponding to the molybdenum foil current collector can be seen. This result supports the observations in the electrochemistry (Figure 1b,c), wherein the electrochemical reduction of Se(0) to Se(−II) can occur only after the initial charge reaction in a crystalline electrode, presenting an additional corollary between the crystalline order and the existence of the Se to Al_2_Se_3_ conversion reaction.

Solid-state ^77^Se NMR spectra were acquired of *t*-Se powder and the composite powder of *a*-Se melted into Ketjen black to observe the differences between *t*-Se and *a*-Se at a molecular level (Figure 3). Both *t*-Se and *a*-Se each contain only a single ^77^Se signal, at 795 and 869 ppm for *t*-Se and *a*-Se, respectively. A single ^77^Se signal indicates that all of the Se environments contained therein are magnetically equivalent. Trigonal selenium has been shown to have two connected intrachain neighbors, and four unconnected interchain neighbors totaling a near-octahedral coordination environment.^21,23^ Several studies have determined that trigonal selenium maintains its chain structure upon melting,^21,24−26^ a conclusion that is further supported by Marple et al., who used solid-state ^77^Se NMR spectroscopy to distinguish between chain-like and ring-like allotropes of selenium.^21^ Since *a*-Se and *t*-Se have similar structures at the atomic scale, the differences in their electrochemical performance must be due to the intermediate-scale structuring, where the trigonal chains of *a*-Se are locally disordered with overlapping chains, as opposed to the aligned chains of *t*-Se.

**Figure 3:**
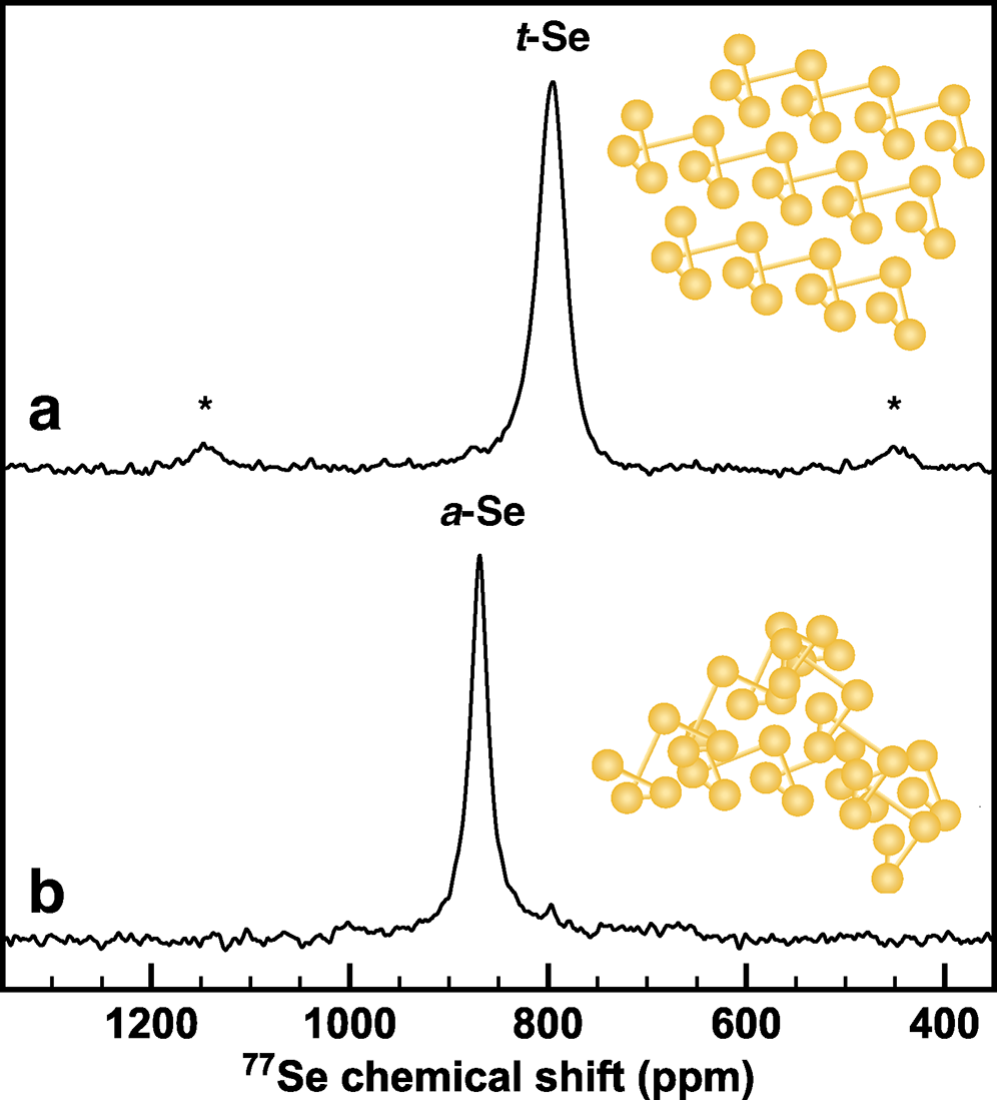
Solid-state ^77^Se NMR spectra comparing (a) *t*-Se powder and (b) *a*-Se composite powder acquired at 14.1 T, 40 kHz, and 25 kHz MAS, respectively. Asterisks (*) denote spinning sidebands. Inset: corresponding selenium structures.

The local structure of Se plays a critical role in its electrochemical response compared to S electrodes. The helical crystalline structure of *t*-Se is fundamentally different compared to the eight-membered puckered-ring structure of S_8_, leading to significant differences, in terms of both atomic environments and crystal packing. The helical *t*-Se structure results in high degrees of secondary bonding interactions that are absent in eight-membered-ring structures.^23^ Note that selenium has a metastable allotrope with an eight-membered puckered ring, α-Se, which has very distinct properties compared to those of *t*-Se. However, previous solid-state ^77^Se NMR studies indicate that *t*-Se is not expected to form Se_8_ rings.^21,27^

*a*-Se has the same local bonding topology compared to *t*-Se but loses its long-range periodic atomic ordering and exhibits variances in these secondary bonding interactions. Chalcogenide glasses have been previously proposed to contain defects known as valence alternation pairs^28,29^ that can lead to dynamic bond formation.^30^ The resultant over- and undercoordinated selenium atoms might serve as sites for electrochemical redox reactions. Clearly, differences in local structure and environment—but not bonding topology—enable the viability of the electrochemical reduction of *a*-Se compared to *t*-Se, giving rise to its favorable electrochemical discharge. Possible topics of interest for future studies would be to investigate this electrochemical reduction process using the α-Se allotrope, as well as using tellurium electrodes,^31^ since tellurium has a chain structure similar to that of selenium and is not known to form eight-membered-ring structures.^23^

Overall, the results show that the Se to Al_2_Se_3_ reaction is obtainable with amorphous selenium, whether achieved incipiently by melt-infusion or indirectly as the reaction products of reversible selenium oxidation reactions, but is scarcely possible for crystalline selenium. These differences were exemplified through electrochemical cycling tests, where the reduction of Se is not observed with fresh *t*-Se electrodes but is possible either in subsequent cycles or in *a*-Se electrodes. Further, the solid-state NMR results reiterate previous findings that the chain structure of *t*-Se is maintained at the molecular level; however, melting incurs a change in the longer-range interactions between molecularly proximate Se chains resulting in a modest ^77^Se chemical shift increase versus *t*-Se (+80 ppm). Here, we avoid complex electrode optimization processes to focus only on the origins of the seemingly capricious electrochemical reduction of Se to Al_2_Se_3_ (Se(0) to Se(−II)), which has been sporadically reported in the literature and often missed. The study of these chalcogen electrodes across multiple length scales highlights the importance of understanding interactions that can be obfuscated when using only highly engineered systems. Therefore, this study explains the discrepancies in the existing aluminum–selenium battery literature and highlights the impacts of selenium crystallinity on achieving the full six-electron capacity of selenium.

## Supporting Information Available

Materials and methods, galvanostatic cycling statistics, X-ray diffraction patterns of pristine Se powders, solid-state ^77^Se NMR of pristine Se powders, galvanostatic cycling of melt-annealed Se before and after potential window increase, scanning electron micrographs of pristine Se powders, galvanostatic charge of Al–Se cell harvested for XRD of charged *t*-Se electrode, XRD patterns of Kapton tape, and pristine and charged *t*-Se electrodes (PDF)
