# Supplementary material for: Elucidating Consequences of Selenium Crystallinity on Its Electrochemical Reduction in Aluminum–Selenium Batteries
Source: ACS Mater Lett. 2024 May 24;6(7):2577–81. doi: 10.1021/acsmaterialslett.4c00531 (PMC11220787; doi:10.1021/acsmaterialslett.4c00531)
Supplement: Supplementary file 1 — tz4c00531_si_001.pdf [file tz4c00531_si_001.pdf]

Supporting Information

Elucidating Consequences of Selenium Crystallinity on its Electrochemical Reduction in Aluminum-Selenium Batteries

Leo W. Gordon†, Rahul Jay†, Ankur L. Jadhav, Snehal S. Bhalekar, Robert J. Messinger*

Department of Chemical Engineering, The City College of New York, CUNY, New York, NY, 10031, USA

*E-mail: rmessinger@ccny.cuny.edu

MATERIALS & METHODS

Electrode Fabrication. Electrodes were prepared using two methods, direct incorporation (*t*-Se) and melt infusion (*a*-Se). Selenium powder (Alfa Aesar, 99%) was mixed with Ketjen Black conductive carbon (Nouryon, 1400 $m^2 g^{-1}$) in a 4:1 weight ratio, for *t*-Se electrodes, this mixture was ball-milled with Super P conductive carbon (Alfa Aesar, 99%) for a total of 20 minutes (4x 5 minutes with 2 minute rests) and combined poly(vinylidene fluoride) (PVDF) binder in an 8:1:1 mass ratio, for a final ratio total of Se:Ketjen Black:Super P:PVDF of 64:16:10:10. For *a*-Se cathodes, the Se:Ketjen Black mixture was heated to 250 °C in a tube furnace for 12 h under flowing argon and quenched in air, otherwise the same procedure as for *t*-Se was followed for electrode fabrication. Melt-annealed Se was prepared with the same process as *a*-Se, but was annealed at 150 °C for 12 h instead of air quenching.

Cell Assembly. Cells were assembled in an argon-filled VAC glovebox with H_2O and O_2 levels below 1 ppm. Poly(tetrafluoroethylene) (PTFE) Swagelok unions of 0.25-in and 0.5-in diameters were used as cell bodies, with molybdenum rods as current collectors. Glass microfiber filters (GF/D Whatman) were used as separators, and aluminum foils (99.99 % Alfa, 0.1-mm thick) were used as anodes. A Lewis acidic mixture of aluminum chloride (Alfa Aesar; 99.99% metals basis) and 1-ethyl-3-methylimidazolium chloride (Aldrich; ≥98.0%) (1.5:1 molar ratio) was used as the electrolyte. For 0.25-in cells, 35 µl of electrolyte was added, for 0.50-in cells 100 µl of electrolyte was added.

Electrochemical Characterization. All potentials here are referenced versus Al/Al(III) redox couple using an aluminum foil pseudoreference that also acts as the counter electrode. Galvanostatic cycling experiments were performed on an Arbin Instruments LBT battery cycler, using lower and upper voltage limits of 0.2 V and 1.5/2.25 V, respectively. Cyclic voltammetry measurements were performed on a 6-channel BioLogic VSP-300 potentiostat from 0.2 to 1.5/2.25 V at a rate of 0.5 mV s^{-1}. All electrochemical characterization was performed at room temperature.

Powder X-ray Diffraction. Powder XRD measurements were performed using a PANalytical X'Pert Pro powder diffractometer with a Cu Kα radiation source (λ = 0.544 nm). A 2θ range of 10–80° was scanned at a rate of 0.05° s^{-1}.

Scanning Electron Microscopy. Electron micrographs were acquired using a Zeiss Supra 55 field emission scanning electron microscope under high vacuum.

Nuclear Magnetic Resonance Spectroscopy. Solid-state NMR spectra were acquired on a Bruker AVANCE III HD 600 NMR spectrometer with a 14.1 T narrow-bore superconducting magnet operating at 114.458 MHz for ^{77}Se. A Phoenix NMR 1.6-mm HXY magic-angle-spinning (MAS) probehead was used. To mitigate MAS-induced sample heating, air at a temperature of 298.1 K was pumped through the probehead at 600 L h^{-1}. ^{77}Se chemical shifts were referenced to *t*-Se (δ_{iso} = 795 ppm) as a secondary ^{77}Se chemical shift reference. MAS rates of 40 kHz and 25 kHz were used for *t*-Se and *a*-Se, respectively. The slower MAS rate was used for the *a*-Se composite powder due to the high conductivity of the Ketjen Black. Solid-state ^{77}Se spin-echo NMR experiments were acquired to eliminate acoustic ring-down effects. Rotor-synchronized half-echo delays (τ/2) of 25 µs (40 kHz MAS) or 40 µs (25 kHz) were used, resulting in full-echo delays of 50 µs or 80 µs, respectively, while data was acquired from the top of the echo. All ^{77}Se pulses used an rf field strength of 108.7 kHz (π/2 of 2.3 µs). Recycle delays of 240 s and 5 s were used for *t*-Se

and *a*-Se, respectively; which were experimentally determined to enable quantitative solid-state ^{77}Se NMR measurements. The solid-state ^{77}See spectrum of crystalline *t*-Se was acquired with 1024 scans (recycle delay = 240 s, 68 h experimental time), the solid-state ^{77}Se spectrum of *a*-Se was acquired with 19456 scans (recycle delay = 5 s, 27 h experimental time). Solid-state ^{77}Se spectra of melt-annealed Se were acquired with either 977 scans (recycle delay = 5 s, 1.4 h experimental time) or 3072 scans (recycle delay = 240 s, 205 h experimental time), where the latter was used for quantitative measurements and the former was used as a longitudinal relaxation filter to suppress crystalline ^{77}Se signals.

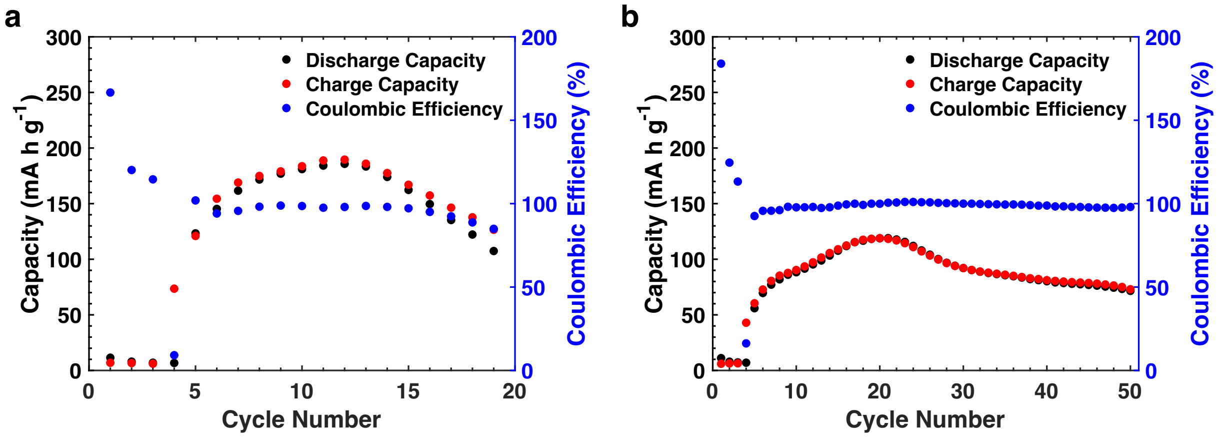

Figure S1. Galvanostatic cycling statistics of (a) the *t*-Se cell shown in Figure 1c, and (b) another *t*-Se cell cycled under the same protocol. Both cells have minimal capacity with a 1.5 V upper cutoff (first 3 cycles), and have steadily increasing capacities after the 3rd cycle, where the upper voltage limit is increased to 2.25 V. This increase is in competition with material dissolution into the electrolyte that reduces the capacity each cycle.

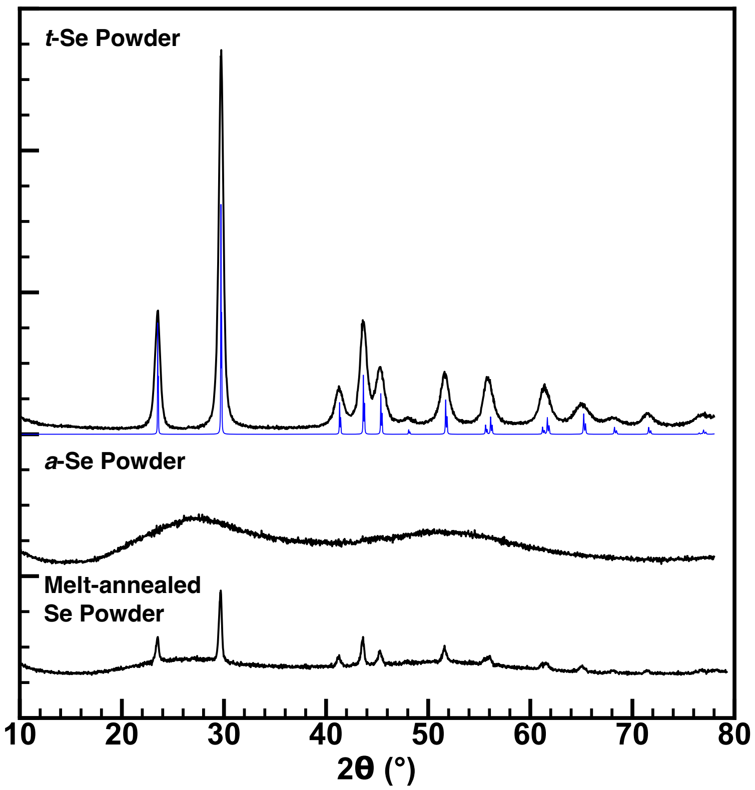

Figure S2. X-Ray diffraction patterns of *t*-Se powder, *a*-Se powder, and melt-annealed Se powder. The simulated diffraction pattern of *t*-Se is given in blue under the experimental pattern.

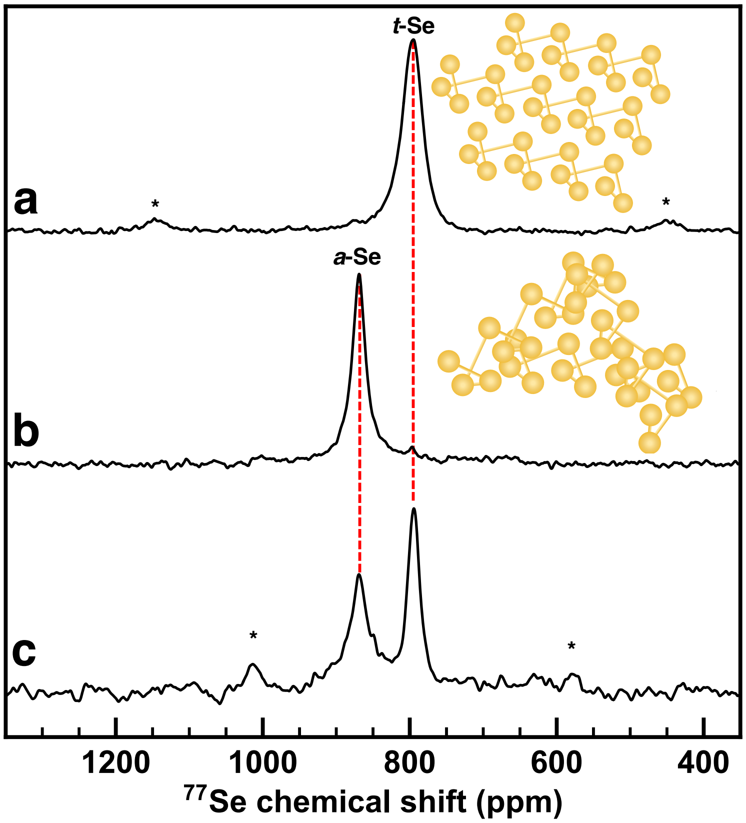

Figure S3. Solid-state ^{77}Se NMR spectra comparing (a) *t*-Se powder, (b) *a*-Se powder, and (c) melt-annealed Se powder, acquired at 14.1 T and 40 kHz, 25 kHz and 25 kHz MAS, respectively. Asterisks (*) denote spinning sidebands, note that (c) only shows spinning sidebands for the crystalline *t*-Se. Inset: corresponding selenium structures. Integration of the two signals in the melt-annealed Se powder suggests the composition is 50.5 mol% *a*-Se and 49.5 mol% *t*-Se.

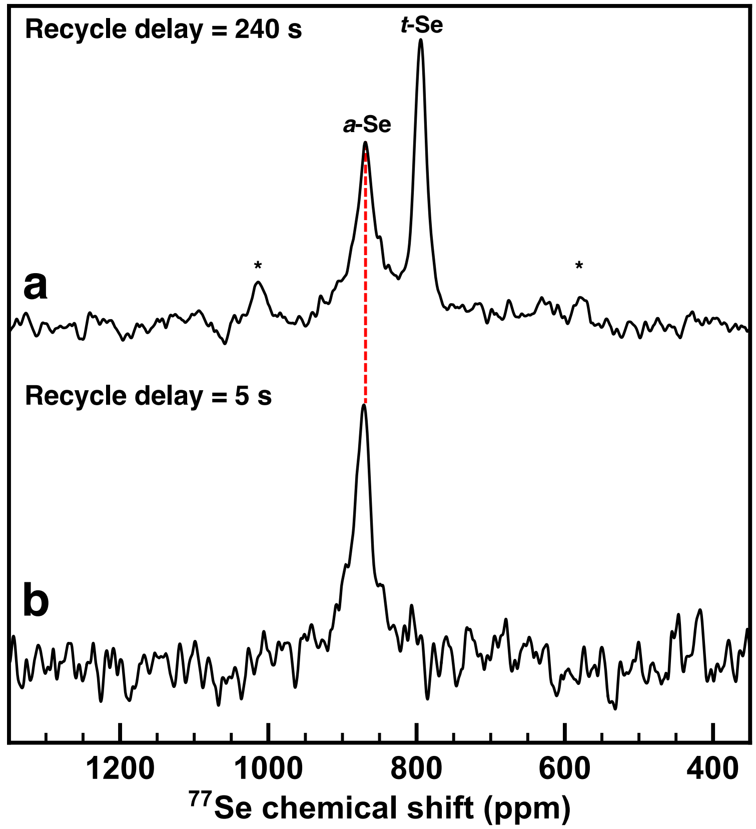

Figure S4. Solid-state ^{77}Se NMR spectra of melt-annealed Se powder with (a) a 240 s recycle delay, and (b) a 5 s recycle delay, acquired at 14.1 T and 25 kHz MAS. Asterisks (*) denote spinning sidebands. Figure S4a is identical to Figure S3c. The 5 s recycle delay in (b) is quantitative for *a*-Se but is too short for *t*-Se, thus fails to capture the *t*-Se signal that is observable in (a).

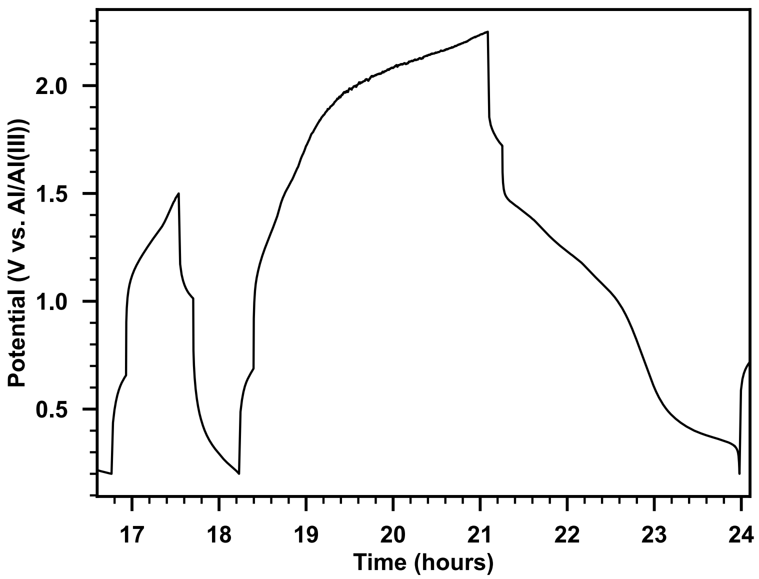

Figure S5. (a) Galvanostatic cycling plot of an Al-Se cell using melt-annealed Se electrodes charged initially to 1.5 V, then to 2.25 V at a rate of 30 mA g^{-1} demonstrating the increase of the Se(0) to Se(–II) reaction capacity immediately following the high-voltage charge-discharge reactions that form *a*-Se. This plot shows capacity of the Se(0) to Se(–II) reaction before the voltage limit increases due to the amorphous material initially present in the melt-annealed sample.

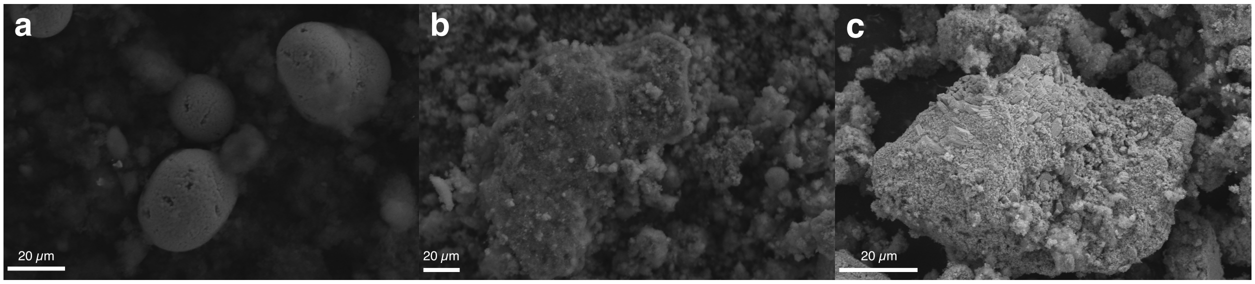

Figure S6. Scanning electron micrographs (SEM) of (a) crystalline *t*-Se powder, (b) amorphous *a*-Se powder melted into Ketjen Black conductive carbon, and (c) melt-annealed Se powder into conductive carbon. In (b) and (c), the large particles are carbon. These SEM images show that the melting process decreases the primary selenium particle size. In (c), note the visible presence of crystalline hexagonal *t*-Se domains.

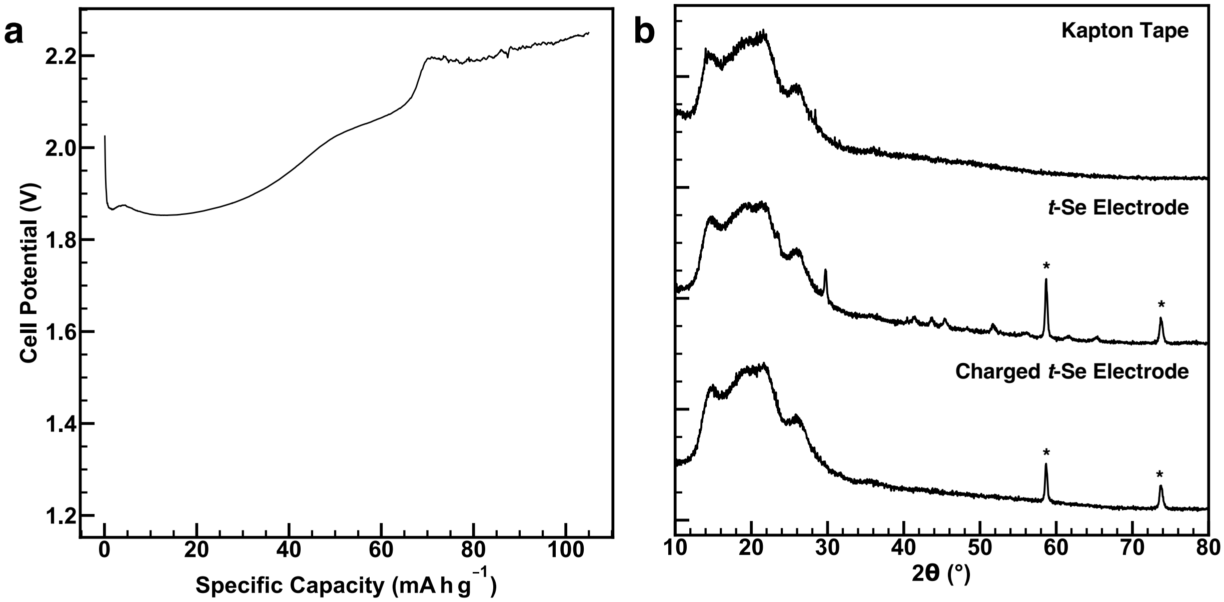

Figure S7. (a) Galvanostatic cycling plot of *t*-Se charged to 2.25 V to harvest the cathode for the XRD measurement in (b). (b) X-Ray diffraction patterns of Kapton tape used for an airtight seal, a pristine *t*-Se electrode, and a *t*-Se electrode charged to 2.25 V.
